# Efficacy and Safety of Topical Therapy With Botanical Products for Melasma: A Systematic Review and Meta-Analysis of Randomized Controlled Trials

**DOI:** 10.3389/fmed.2021.797890

**Published:** 2022-01-24

**Authors:** Tianyun Wang, Youmei Wang, Jue Wang, Hongwei Chen, Biao Qu, Zheng Li

**Affiliations:** ^1^Department of Endocrinology, Huaian Hospital, Huaian, China; ^2^Department of Pharmacy, Huaian Hospital, Huaian, China; ^3^State Key Laboratory of Quality Research in Chinese Medicines, Macau University of Science and Technology, Macau, China; ^4^School of Public Health, Sun Yat-sen University, Shenzhen, China; ^5^Jiangsu Engineering Research Center of Cardiovascular Drugs Targeting Endothelial Cells, School of Life Sciences, College of Health Sciences, Jiangsu Normal University, Xuzhou, China; ^6^State Key Laboratory of Natural and Biomimetic Drugs, Peking University, Beijing, China

**Keywords:** botanical products, topical therapy, melasma, efficacy, safety, systematic review, meta-analysis

## Abstract

Botanical products have been increasingly popular in topical therapies for melasma, as presumed safer and milder than fully synthetic products. Although the efficacy of different topical botanicals has recently been substantiated through randomized controlled trials (RCTs), there is a lack of sufficiently pooled evidence on their efficacy and safety for the treatment of melasma. Herein, a systematic review and meta-analysis was conducted on the efficacy and safety of topical botanical products for the treatment of melasma, following Preferred Reporting Items for Systematic Reviews and Meta-Analyses (PRISMA). All RCTs on the use of topical botanical products for the treatment of melasma in humans were included, except for trials enrolling pregnant patients. The primary outcome was Melasma Area and Severity Index (MASI) or its variation. The secondary outcomes included Mexameter^®^ reading, melasma improvement evaluated by participants, and any reported adverse events (AEs). As a result, twelve eligible trials comprising 695 patients with melasma from 6 different countries were included. The topical botanical products contained active ingredients which varied among trials as follows: herb-derived molecule, extracts of a single herb, and extracts of compound herbs. Topical therapy with botanical products significantly improved melasma with a large effect on MASI reduction (SMD −0.79, 95% CI −1.14 to −0.44, *p* < 0.00001), and a moderate effect on Mexameter^®^ reading reduction (SMD −0.52, 95% CI −0.81 to 0.23, *p* = 0.0005), when compared with placebo. It also showed a similar improvement of melasma with a better safety profile (RR 0.37, 95% CI 0.15–0.88, *p* = 0.02), when compared with active-comparators. Botanical products were well-tolerated across studies, with no serious AEs reported. Despite the limitations such as small sample size, short duration of follow up and varied botanical products, this work still represents the best level of evidence currently available on topical use of botanical products on melasma. Moreover, it should be noted that more well-designed studies are needed before recommending topical botanical products as a viable treatment option for melasma.

**Systematic Review Registration:**

PROSPERO, identifier: CRD42021256328.

## Introduction

Melasma is an acquired hyperpigmentation disorder characterized by the appearance of abnormal melanin deposits in different layers of skin, especially the face and neck (1). Although melasma can occur in both sexes and any skin type, it has a high prevalence in adult women with darker complexions, especially those living in areas with intense sun exposure (2–4). As classified according to the location of the pigment, three types of melasma exist, namely, epidermal, dermal, and mixed types (5). Although its pathogenesis has not yet been fully elucidated, genetic factors, chronic ultraviolet (UV) exposure, and hormones have been demonstrated to be implicated in the occurrence of it (6–8). Recent studies also suggested the role of inflammatory processes in the pathogenesis of melasma (2, 9).

The physiological and psychological effects of melasma have a considerable negative impact on the quality of life of affected individuals. This distressing condition is exacerbated by the therapeutic challenge due to its refractory and recurrent nature (3, 10). To date, various therapy modalities have been developed. Topical therapy with photoprotection and lightning products is still the mainstay for the treatment of melasma. Among these products, hydroquinone (HQ) has been used as the benchmark for decades, especially in epidermal melasma (3). It is a competitive inhibitor of tyrosinase which prevents the enzymatic oxidation of tyrosine to dopa, thus, preventing the synthesis of melanin. Unfortunately, safety concerns surrounding HQ are still controversial. The adverse events (AEs) were reported as exogenous ochronosis and permanent depigmentation (11). Other safety issues regarding its systemic absorption and drug-induced carcinogenesis have also been expressed (12, 13). Second-line treatment options include oral tranexamic acid, lasers, and chemical peels. However, no consensus has been reached on their robust efficacy for melasma, let alone the accompanied AEs, namely, postinflammatory dyspigmentation, scarring, and venous thromboembolism (14).

Botanical extracts have been used empirically in topical therapy for different diseases since ancient times. During past decades, many herbal extracts or isolated molecules have been reported to show the activities of inhibiting tyrosinase, scavenging free radicals, and suppressing inflammatory processes (15–17). Some of these have been used in topical drugs or cosmetic formulations for the treatment of melasma. These botanical products are increasingly popular as they are presumed safe, mild, and available over the counter (18). Only recently, though, the efficacy of some botanical products has been substantiated through clinical trials (3). In these trials, Melasma Area and Severity Index (MASI) is widely adopted as a standardized subjective method for evaluating efficacy (19). In addition, subjective methods are now often supplemented with objective methods, such as spectroscopic analysis using a Mexameter^®^. Although different topical botanical products have been evaluated for the treatment of melasma in many randomized controlled trials (RCTs), there is a lack of sufficiently pooled evidence on their efficacy and safety. In an attempt to address this uncertainty, we conducted a systematic review and meta-analysis of RCTs to investigate the efficacy and safety of topical botanical products in patients with melasma.

## Materials and Methods

This systematic review and meta-analysis was prospectively registered at the International Prospective Register of Systematic Reviews (PROSPERO) (registration number. CRD42021256328) and conducted following the Preferred Reporting Items for Systematic Reviews and Meta-Analyses (PRISMA) guidelines (20).

### Search Strategy

A systematic search was carried out using PubMed, Embase, the Cochrane Library, and Web of Science to collect relevant studies from inception to September 8, 2021. This search was conducted by two independent reviewers (BQ and HC), and the complete search strategies were reported in Supplementary Table 1. Reference lists of included studies and relevant systematic reviews were also reviewed manually to find additional eligible studies.

### Study Selection

All identified studies were assessed to determine whether they met the following criteria: RCTs investigating the efficacy and safety of botanical products used in topical therapies for the treatment of melasma in humans. We excluded studies that included participants with pregnancy or breastfeeding. Moreover, participants included in trials should be healthy adults with melasma diagnosed by dermatological consultation and/or device examination. The first screening for potentially relevant records based on title and abstract, and following eligibility assessment based on full-text, were independently conducted by two reviewers (TW and YW). Any disagreement between investigators was resolved by discussion to reach a consensus.

### Outcome Measures

The primary outcome was the improvement in melasma severity evaluated through the changes of MASI or its variation from baseline to follow-up. The secondary outcomes included melasma improvement evaluated through the changes of Mexameter^®^ reading, improvement evaluated by participants, and any reported AE.

### Data Extraction

Baseline characteristics and outcome data were extracted independently by two authors (TW and YW) using a standard data extraction form, with disagreements resolved by consensus. The following information from each study was extracted: the surname of the first author, publication year, country of origin, study period, study design, number of participants, percentage of female participants, mean ages of participants, description of interventions, and outcome measures. Data of multiple groups from one study were extracted using the recommendations from the Cochrane Handbook (21). For continuous outcomes, the following were extracted: means, SD, and sample sizes at baseline and follow-up. For dichotomous outcomes, the number of cases and total sample sizes were extracted.

### Quality Assessment

The risk of bias was assessed independently by two investigators (TW and YW) using the Cochrane Risk of Bias Tool (21) with disagreements resolved by discussion to reach a consensus. Based on the risk of bias, the included RCTs were graded as low, moderate, or high quality following the criteria (22): (1) RCT was considered low quality if either randomization or allocation concealment was considered at high risk; (2) RCT was considered high quality when both randomization and allocation concealment were considered at low risk, and all other items were considered at low or unclear risk; and (3) RCT was considered moderate quality if it met neither the criteria for high nor low quality.

### Data Synthesis and Analysis

Data collected from RCTs were preprocessed in Microsoft Excel before meta-analyses were performed using RevMan (Ver. 5.4) using random effects. Pooled continuous data were expressed as standardized mean difference (SMD) and pooled dichotomous data were expressed as risk ratio (RR), with 95% CI. To facilitate the interpretation of estimated efficacy, we interpreted pooled SMD using rules of thumb as follow: <0.40 = small effect, 0.40–0.70 = moderate effect, >0.70 = large effect (21).

In this study, the meta-analyses compared: (1) efficacy of topical therapy with botanical products at each time point; (2) efficacy of topical therapy with botanical products compared with placebo; and (3) efficacy of topical therapy with botanical products compared with active-comparators. Therefore, subgroup analyses were conducted in these cases by intervention duration and comparator type (placebo/active-comparator). Heterogeneity was assessed through the I^2^ statistic. For *I*^2^ statistics, a value of <30, 30–60, and >60% represented low, moderate, or high heterogeneity, respectively. A *p* < 0.05 was considered significant for the test for the overall effect. Funnel plots were assessed to detect potential small-study effects as a signal of publication bias.

## Results

### Search Results

**Figure 1:**
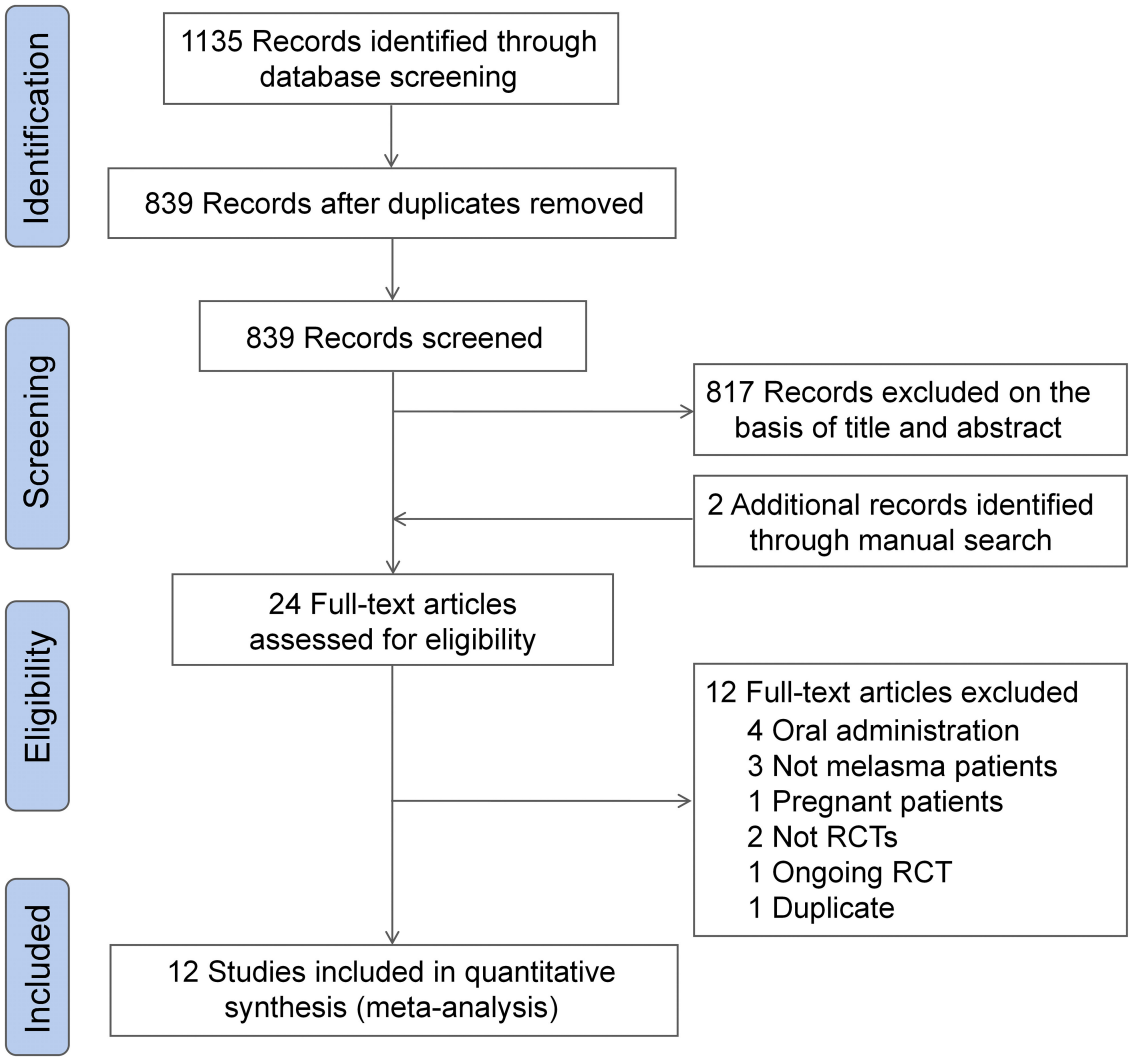
Flow diagram representing the study selection process.

The initial literature search yielded 839 unique records. A total of 817 records were excluded after the first screening, and two additional records were retrieved through a manual citation and reference search of relevant articles. The full-text of 24 studies were reviewed for inclusion, and finally, 12 eligible RCTs were included for meta-analysis (23–34). The detail of the study selection process are shown in Figure 1.

### Study Characteristics

Characteristics of included RCTs were summarized in Table 1. These 12 studies included 695 patients with melasma from six different countries, in which female patients accounted for over 80%. Of 12 RCTs included, 2 studies included only epidermal melasma (29, 34), three studies included epidermal and mixed melasma (23, 25, 31), 1 study included only mixed melasma (26), 1 study included all types of melasma (24), and five studies did not specify the types (27, 28, 30, 32, 33). The active ingredients contained in botanical products varied among trials as follows: herb-derived molecules, extracts of a single herb, and extracts of compound herbs. The topical formulations of botanical products used for intervention included cream, oil, brewed, and solution. Six trials used placebo as a comparator (23, 24, 26–28, 32), four trials used actives as a comparator (25, 29, 30, 34), and two trials used both placebo and actives as a comparator (31, 33). The duration of intervention ranged from 4 to 12 weeks with a maximum follow-up duration of 16 weeks. A total of seven trials reported the incidence of AEs (23, 25, 29–31, 33, 34). Of 12 RCTs included, four declared sponsorship from non-profit organizations (24, 28–30), three from commercial industries (25, 27, 33), and five did not declare sponsorship (23, 26, 31, 32, 34).

**Table 1:** Characteristics of included studies.

**Study**	**Country**	**Sample size (% female) and characteristics**	**Interventions**	**Duration (weeks)**	**Outcomes**	**Adverse events**
Alvin et al. (23)	Philippines	Size: 50 (98%) Age: 44.5 ± 7.5 (25–60) Melasma type: epidermal, mixed Melisma duration: 0–5 years	Oil (Mulberry) ***Vs***. Placebo	8	MASI Mexameter reading Self-evaluation	**EG**: 16% 4 × mild itching **CG**: 48% 8 × mild pruritus 4 × mild erythema
Bavarsad et al. (24)	Iran	Size: 22 (100%) Age: 34.10 ± 8.99 Melasma type: epidermal, dermal, mixed Melisma duration: 210 ± 118 weeks	Cream (Lycopene and Wheat bran) ***Vs***. Placebo	12	MASI Discoloration rate Area of melasma Self-evaluation	0%
Costa et al. (25)	Brazil	Size: 56 (100%) Age: 18–60 Melasma type: epidermal, mixed Melisma duration: NR	Cream (Emblica, Licorice and Belides) ***Vs***. Cream (2% HQ)	12	Medical evaluation Self-evaluation UV spots Manchas UV	**EG**: 8.7% 2 × burning & increase of previous acne lesions **CG**: 26.9% 7 × erythema & burning & erythematous papules in the perioral region
Francisco-Diaz et al. (26)	Philippines	Size: 52 (84.6%) Age: 18–60 Melasma type: mixted Melisma duration: 3.75 years (mean)	Solution (Malva sylvestris, Mentha piperita, Primula veris, Alchemilia vulgaris, Achillea millefolium, Mellissa officinales) ***Vs***. Placebo	12	mMASI Area of melasma Light reflectance	0%
He et al. (27)	China	Size: 70 (100%) Age: 24–52 Melasma type: NR Melisma duration: 0.5–20 years	Cream (herbal medicines) ***Vs***. Placebo	8	MASI Medical evaluation	0%
Javedan et al. (28)	Iran	Size: 60 (81.6%) Age: 32.18 ± 8.69 (26–55) Melasma type: NR Melisma duration: NR	Cream (Dorema ammoniacum) ***Vs***. Placebo	4	mMASI	0%
Khosravan et al. (29)	Iran	Size: 70 (100%) Age: 19–55 Melasma type: epidermal Melisma duration: NR	Parsley brewed ***Vs***. Cream (4% HQ)	8	MASI	**EG**: 7.4% 2 × redness & itching **CG**: 7.4% 2 × redness & itching
Mahjour et al. (30)	Iran	Size: 40 (100%) Age: 18–59 Melasma type: NR Melisma duration: NR	Cream (C. Aritinum L. and C. melo var. inodorus H.Jacq) ***Vs***. Cream (4% HQ)	12	MASI Self-evaluation	**EG**: 3.1% 1 × acne **CG**: 15.6% 3 × erythema 1 × ecne 1 × erythema & dryness & scaling
Mendoza et al. (31)	Philippines	Size: 45 (62.2%) Age: 29.04 ± 7.8 (18–50) Melasma type: epidermal, mixed Melisma duration: NR	Cream (Rumex occidentalis) ***Vs***. 1. Cream (4% HQ) 2. Placebo	8	MASI Mexameter reading Medical evaluation Self-evaluation	**EG**: 6.7% 1 × mild peeling **CG1**: 0% **CG2**: 0%
Morag et al. (32)	Poland	Size: 50 (100%) Age: 37.67 ± 7.53 (26–55) Melasma type: NR Melisma duration: NR	Cream (Five-leaf serratula) ***Vs***. Placebo	8	Mexameter reading	0%
Zhang et al. (33)	China	Size: 90 (NR) Age: 40.35 ± 6.02 (25–50) Melasma type: NR Melisma duration: 5.46 ± 3.72 years	Cream (China camellia, sanchi, Prinsepia utilis oil, and Portulaca oleracea) ***Vs***. 1. Cream (arbutin) 2. Placebo	12	MASI Mexameter reading Inflammatory cells Self-evaluation	**EG**: 0% **CG1**: 6.7% 2 × slight erythema and pruritus **CG2**: 0%
Zubair et al. (34)	Pakistan	Size: 90 (96.7%) Age: 29.31 ± 6.47 (18–40) Melasma type: epidermal Melisma duration: 5.80 ± 3.93 years	Cream (4% Liquiritin) Cream (2% Liquiritin) ***Vs***. Cream (4% HQ)	8	Medical evaluation	**EG**: 0% **CG**: 10% 2 × contact dermatitis 1 × hyperpigmentation

### Quality Assessment

**Figure 2:**
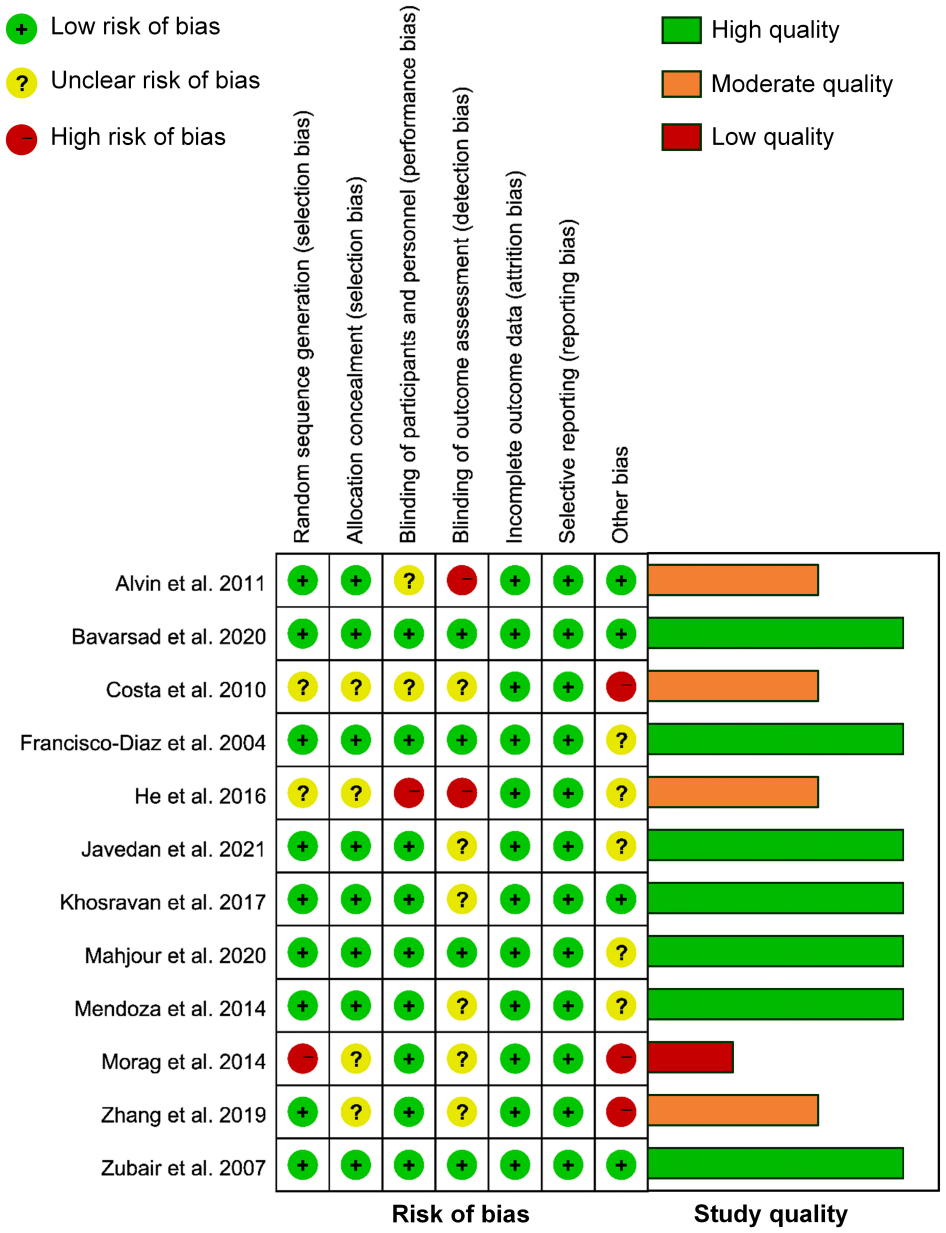
Risk of bias and study quality assessment of included randomized controlled trials (RCTs).

Figure 2 shows the detailed assessment of the risk of bias. Of 12 RCTs included, 1 claimed unblinded (27), 2 claimed single-blind (23, 25), 8 claimed double-blind (24, 26, 28, 29, 31–34), and 1 claimed triple-blind (30). Five studies arose concern for risk of bias for the following reasons (23, 25, 27, 32, 33): (1) one study used an odd-even randomization method; (2) two studies had insufficient information about randomization method; (3) four studies had insufficient information on allocation concealment; (4) three studies did not use adequate blind method; and (5) three studies were funded by commercial industries. According to the declared criteria, seven studies were considered high quality (24, 26, 28–31, 34), four studies were considered moderate quality (23, 25, 27, 33), while one study was considered low quality (32). Overall, the major potential source of high bias was in the “other” bias domain which could be attributed to sponsorship from companies.

### Primary Efficacy Outcomes

**Figure 3:**
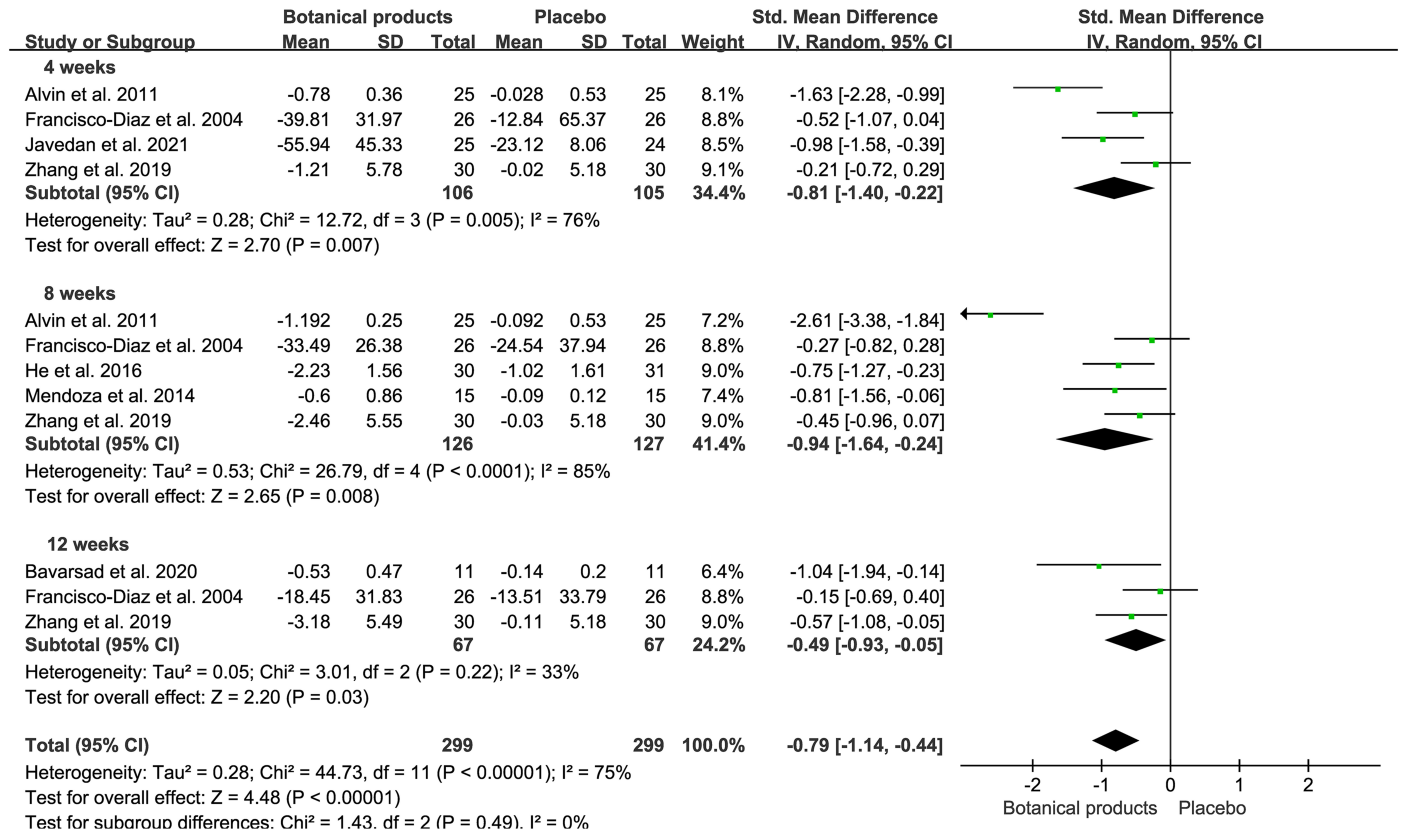
Forest plots depicting the standardized mean difference (SMD) of Melasma Area and Severity Index (MASI) reduction in patients with melasma receiving botanical products in placebo-controlled trials. Subgroup analysis was stratified according to the duration of the intervention.

**Figure 4:**
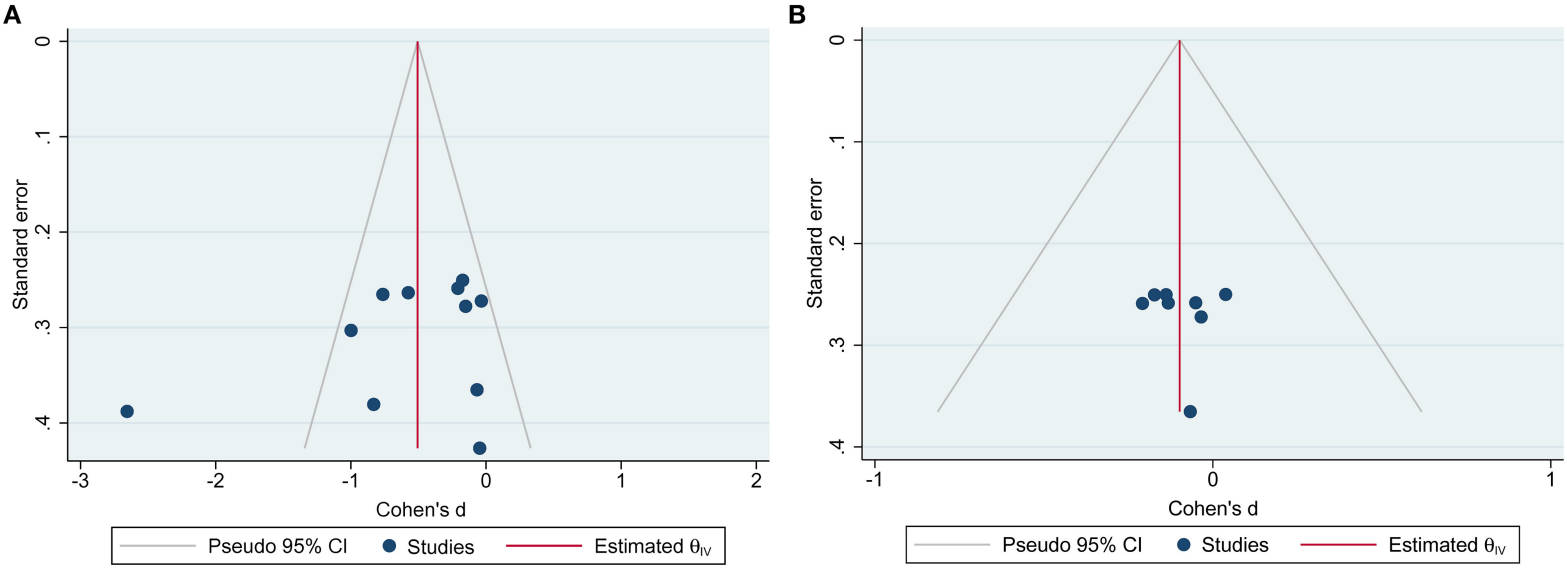
Funnel plots for RCTs reported Melasma Area and Severity Index (MASI) outcome measures. **(A)** Funnel plot for placebo-controlled trials; **(B)** funnel plot for active-controlled trials.

Ten RCTs with MASI outcomes included 299 and 299 participants in the botanical product and placebo groups, respectively. Pooled results showed that botanical products had a large effect on MASI reduction vs. placebo at 4 weeks (SMD −0.81, 95% CI −1.40– −0.22, *p* = 0.007; Figure 3). The effect size was larger when assessed at 8 weeks (SMD −0.94, 95% CI −1.64– −0.24, *p* = 0.008), compared with 4 weeks. However, the smallest effect was seen at 12 weeks (SMD −0.49, 95% CI −0.97– −0.05, *p* = 0.03). Therefore, the MASI reduction in patients receiving topical botanical products vs. placebo did not amplify with treatment time. Overall, botanical products improved melasma with a large effect vs. placebo (SMD −0.79, 95% CI −1.14– −0.44, *p* < 0.00001). In view of high heterogeneity across studies, we conducted a sensitivity analysis using fixed-effects, but the overall results were almost identical (SMD −0.69, 95% CI −0.86– −0.53, *p* < 0.00001). The funnel plot displayed a tolerably symmetrical funnel shape (Figure 4A), and the Egger test (*p* = 0.2919) also revealed the low risk of publication bias.

**Figure 5:**
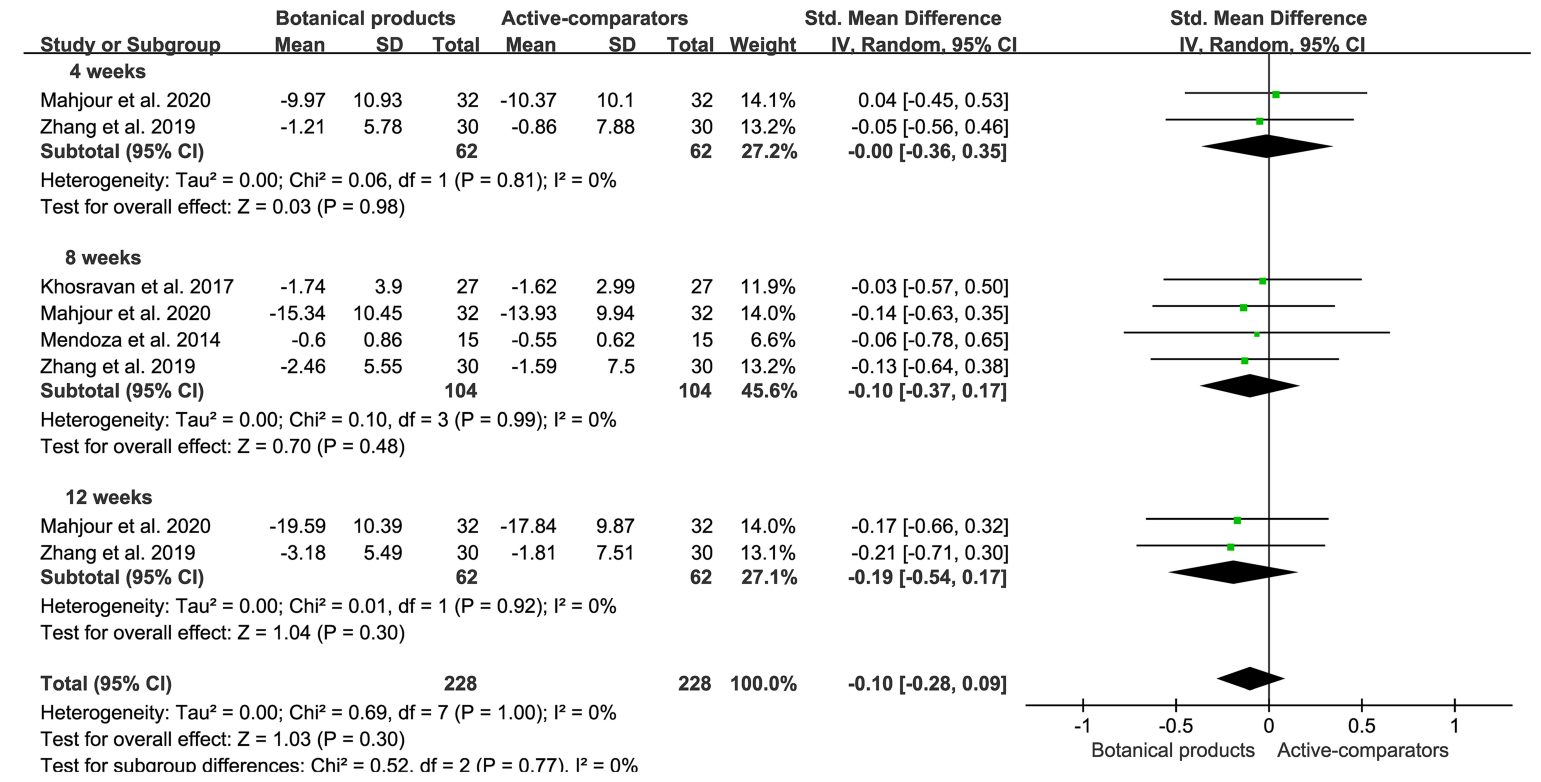
Forest plots depicting the SMD of MASI reduction in patients with melasma receiving botanical products in active-controlled trials. Subgroup analysis was stratified according to the duration of the intervention.

Eight RCTs included 228 and 228 participants in the botanical product and active-comparator groups, respectively. Pooled data showed that the SMD of MASI reduction in patients with botanical products vs. actives was −0.00 (95% CI −0.36–0.35, *p* = 0.98) at 4 weeks, −0.10 (95% CI −0.37–0.17, *p* = 0.48) at 8 weeks, and −0.19 (95% CI −0.54–0.17, *p* = 0.30) at 12 weeks (Figure 5). The overall SMD was −0.10 (95% CI −0.28–0.09, *p* = 0.30), suggesting that botanical products had a similar effect to active-comparators. There was no remarkable asymmetry in the funnel plot (Figure 4B), and the Egger's test (*p* = 0.9167) also suggested the small potentiality of publication bias.

### Secondary Efficacy Outcomes

**Figure 6:**
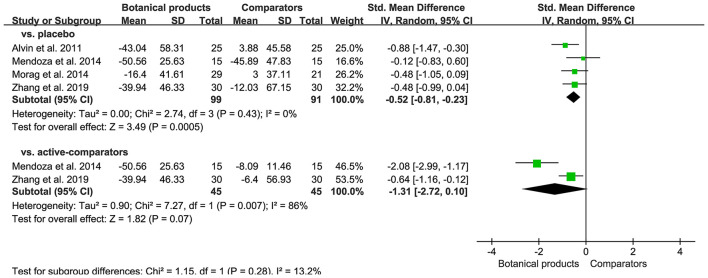
Forest plot depicting the SMD of Mexameter^®^ reading reduction in patients with melasma receiving botanical products in RCTs. Subgroup analysis was stratified according to comparators.

Four placebo-controlled trials containing 190 participants, and active-controlled trials containing 90 participants, reported the efficacy of botanical products through measuring the changes of Mexameter^®^ reading. Meta-analyses showed that botanical products had a moderate effect on the reduction of Mexameter^®^ reading compared with placebo (SMD −0.52, 95% CI −0.81–0.23, *p* = 0.0005), but no significant difference when compared with active-comparators (SMD −1.31, 95% CI −2.72–0.10, *p* = 0.07; Figure 6). There was no heterogeneity between these placebo-controlled trials, but high heterogeneity between these active-controlled trials, and too few studies to assess for publication bias.

Improvement evaluated by patients was reported in 6 RCTs. For placebo-controlled trials, 36 (80.0%) of 45 patients allocated to botanical products reported improvement, compared with 12 (26.7%) of 45 patients receiving placebo, showing significant difference (RR 2.90, 95% CI 1.59–5.29, *p* = 0.0005; Supplementary Figure 1). For active-controlled trials, 86 (86.0%) of 100 patients allocated to botanical products achieved improvement, compared with 78 (75.7%) of 103 patients receiving placebo, but with no significant difference (RR 1.07, 95% CI 0.87–1.32, *p* = 0.50; Supplementary Figure 1).

### Safety Outcome

**Figure 7:**
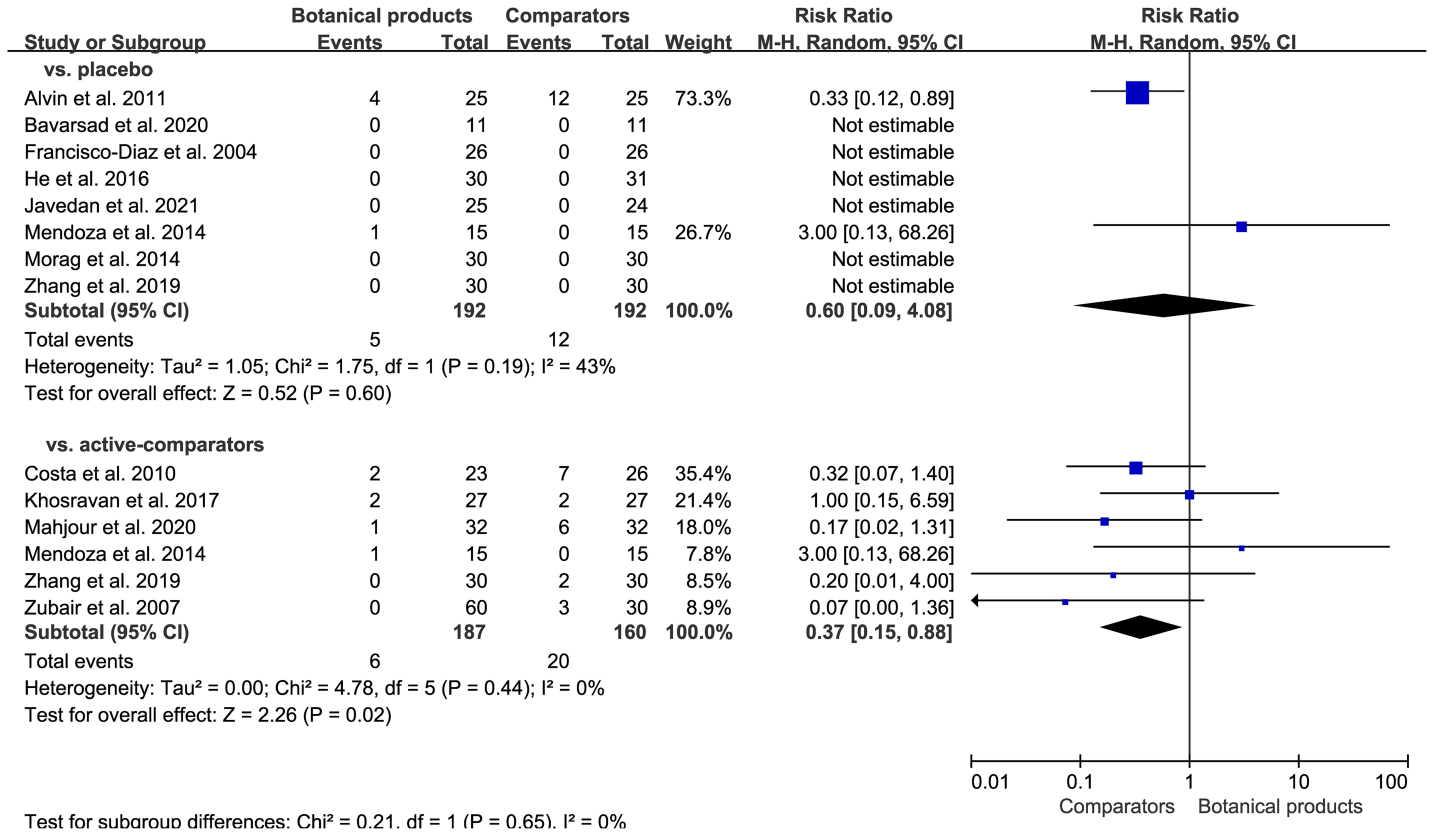
Forest plot depicting the risk ratio (RR) for safety outcome in RCTs investigating the efficacy of botanical products. Subgroup analysis was stratified according to comparators.

Seven of 12 RCTs reported AEs, but no serious AEs. Common AEs included mild itching, erythema, and pruritus. For placebo-controlled trials, there were 5 (2.6%) of 192 patients receiving botanical products experienced AEs, compared with 12 (6.3%) of 192 patients receiving placebo. Pooled data showed that the incidence of AEs in patients taking botanical products was similar to those taking placebo (RR 0.60, 95% CI 0.09–4.08; *p* = 0.60; Figure 6). For active-controlled trials, there were only 6 (3.2%) of 187 patients receiving botanical products experienced AEs, compared with 20 (12.5%) of 160 patients receiving active-comparators. Pooled data also demonstrated a significant reduction in the incidence of AEs in patients receiving botanical products compared with active-comparators (RR 0.37, 95% CI 0.15–0.88, *p* = 0.02; Figure 7). Overall, topical therapy with botanical products was well-tolerated across studies. There was no remarkable asymmetry in the funnel plot (Supplementary Figure 2), and the Egger's test (*p* = 0.2421) also suggested the small potentiality of publication bias.

## Discussion

To the best of our knowledge, this systematic review and meta-analysis could be the first to comprehensively assess the efficacy and safety of botanical products for the treatment of melasma. The pooled results suggested that botanical products significantly improved melasma compared to placebo and showed comparable efficacy to active-comparators. Currently, the most common subjective outcome is MASI, as confirmed by that 9 of 12 included RCTs adopted it. The effect sizes of botanical products for MASI outcomes were large compared with placebo. Objective outcome measures were used in some studies, such as Mexameter^®^ reading, which was adopted in 4 of 12 included RCTs. Our meta-analysis demonstrated that there was significant benefit associated with the use of botanical products in Mexameter^®^ reading when compared with placebo, and no difference when compared with active-comparators. These results were consistent with the findings from pooled MASI outcomes. In addition, our systematic review demonstrated that botanical products were well-tolerated, with only a small proportion of patients experiencing mild AEs. Our meta-analysis also suggested that rates of AEs were lower for botanical products compared to active-comparators and were comparable to placebo.

Although topical phytotherapy produced a significant MASI reduction, we noted a decrease in effect size at the 12th week when compared with the 4th and 8th weeks. This condition was in agreement with the results from one trial where MASI reduction was reversed at the 12th week compared with the 8th week (26). These data suggested that the efficacy of topical botanical products might not amplify with treatment time, and even there was a potential to fade. Therefore, their long-time efficacy still needs to be studied and validated in further trials. Moreover, melasma is known to have a high relapse rate which makes efficacy maintenance challenging (3). For instance, in a trial with 12 weeks follow-up, patients reported a melasma relapse with retrogressed MASI within 4 weeks of HQ cessation (35). By contrast, in one of the included trials, a cream containing lycopene and wheat bran caused a significant decrease in MASI during 12 weeks of treatment, with no recurrence within 4 weeks of phytotherapy cessation (24). This could provide a clue that botanical products might have an advantage over HQ in efficacy maintenance, but it still needs to be validated by better studies with a large sample size and longer follow-up.

Melanin is produced from the melanosomes and transferred from melanocytes to keratinocytes (36–38). Melanin synthesis is a tyrosinase-dependent process and requires several oxidative reactions, which consist of tyrosine hydrolysis to dopa, dopa oxidation to quinine, and quinine oxidation to melanin. Transfer of melanosomes to keratinocytes is controlled by keratinocyte protease-activated receptor-2 (PAR-2). The mechanisms of action supporting the efficacy of botanical products in the treatment of melasma vary with the contained active ingredients. Many botanical ingredients bind to the active site of tyrosinase and exert inhibitory activity, which is comparable to HQ (39–41). Moreover, recent studies demonstrate the indirect inhibitory effects of several botanicals on tyrosinase occurring at the transcriptional level through decreasing mRNA expression of tyrosinase-related protein 1 (TRP-1), TRP-2, and microphthalmia-associated transcription factor (MITF) (42, 43). Several botanicals suppress melanosome transfer through inhibition of the keratinocyte PAR-2 (44, 45). Since inflammatory mediators and radicals contribute to melanocyte pigment production, many botanicals inhibit melanin synthesis through anti-inflammatory and antioxidant effects (46, 47). Among the included RCTs, topical therapies with botanical products alleviated melasma through either (1) direct or indirect inhibition of tyrosinase to suppress melanogenesis (29–32); (2) antioxidation to scavenge free radicals (27); (3) regulating inflammatory mediators to inhibit inflammation (28); or (4) synergistic action of above several mechanisms (25, 33, 34).

This meta-analysis had some limitations that merit discussion. First, only a very few studies are RCTs with placebo or active-comparator among numerous published articles, thus the sample size and number of included studies for each meta-analysis were relatively small. Second, the treatment durations were relatively short within 12 weeks, which meant that the long-term efficacy and safety could not be evaluated. Furthermore, of all RCTs included, only one trial that was considered high quality based on the risk of bias assessment, reported the post-treatment assessment with a short follow-up (4 weeks) (24). However, given the recurrent nature of melasma, sufficient trials including a longer duration of posttreatment assessment are indispensable. The third limitation was the information absence on the types of melasma in many RCTs, making it fail to determine the inter-study variability in the type of melasma. A final limitation was the high heterogeneity observed in several meta-analyses, and it is to be expected due to the different botanicals across studies. This was also the uppermost limitation because different botanical products including different compounds and formulations could present different action mechanisms for the treatment of melasma, thus these heterogeneous medications could partly weaken the significance and reliability of obtained findings herein. Nevertheless, this work still represents the best level of evidence currently available on the topical use of botanical products in the management of melasma.

## Conclusion

Botanical products have been increasingly popular in topical therapies for melasma, and many RCTs have been conducted to evaluate their efficacy. However, it still lacks sufficient pooled evidence on their efficacy and safety. Therefore, we conducted a systematic review and meta-analysis on the efficacy and safety of topical botanical products for the treatment of melasma. The pooled results suggested that topical therapy with botanical products produced significant improvement in melasma with beneficial effects on MASI reduction and Mexameter^®^ reading reduction when compared with placebo. It also showed that botanical products produced comparable efficacy for melasma when compared with active-comparators. Moreover, these botanical products were well-tolerated across studies, with no serious AEs reported. This work could represent the best level of evidence currently available on the efficacy and safety of topical botanical products for the treatment of melasma. However, the limitations that existed in this work, namely, small sample size, a short period of treatment, lack of post-treatment follow-up, and importantly heterogeneous medications, could weaken the significance and reliability of these results to some extent. Therefore, it should be noted that more high-quality RCTs with longer intervention and follow-up duration are required before recommending topical botanical products as a viable clinical treatment option for melasma.

## Data Availability Statement

The original contributions presented in the study are included in the article/Supplementary Material, further inquiries can be directed to the corresponding authors.

## Author Contributions

ZL and BQ contributed to the conceptualization and supervision. ZL, TW, and YW contributed to the writing. JW and HC contributed to the software, methodology, and data curation. TW and YW contributed to the investigation and formal analysis. All authors contributed to the article and approved the submitted version of the manuscript.

## Funding

This study was supported by the National Natural Science Foundation of China (Grant No. 82104525), the Natural Science Foundation of the Jiangsu Higher Education Institutions of China (Grant No. 21KJB360009), Natural Science Foundation of Jiangsu Normal University (Grant No. 20XSRX002), and State Key Laboratory of Natural and Biomimetic Drugs (Grant No. K202114).

## Conflict of Interest

The authors declare that the research was conducted in the absence of any commercial or financial relationships that could be construed as a potential conflict of interest.

## Publisher's Note

All claims expressed in this article are solely those of the authors and do not necessarily represent those of their affiliated organizations, or those of the publisher, the editors and the reviewers. Any product that may be evaluated in this article, or claim that may be made by its manufacturer, is not guaranteed or endorsed by the publisher.

## Abbreviations

MASI: Melasma Area Severity Index
EG: experimental group
CG: control group
mMASI: modified MASI
NR: not reported
HQ: hydroquinone.
