# Supplementary material for: Efficacy and Safety of Topical Therapy With Botanical Products for Melasma: A Systematic Review and Meta-Analysis of Randomized Controlled Trials
Source: Front Med (Lausanne). 2022 Jan 24;8:797890. doi: 10.3389/fmed.2021.797890 (PMC8819825; doi:10.3389/fmed.2021.797890)
Supplement: Supplementary file 1 [file Data_Sheet_1.docx]

Supplementary Material

**Supplementary Table 1** Search strategies

| **Pubmed** | |
| --- | --- |
| 1 | "Melanosis"[MeSH Terms] |
| 2 | "melasma*"[Title/Abstract] |
| 3 | "chloasma*"[Title/Abstract] |
| 4 | "freckle*"[Title/Abstract] |
| 5 | "Melanoses"[Title/Abstract] |
| 6 | "Melanism"[Title/Abstract] |
| 7 | #1 OR #2 OR #3 OR #4 OR #5 OR #6 |
| 8 | "Plants, Medicinal"[Mesh] |
| 9 | "Herbal Medicine"[Mesh] |
| 10 | "Drugs, Chinese Herbal"[Mesh] |
| 11 | "Phytotherapy"[Mesh] |
| 12 | "Plant Extracts"[Mesh] |
| 13 | herb*[Title/Abstract] |
| 14 | plant[Title/Abstract] OR plants[Title/Abstract] |
| 15 | botanical[Title/Abstract] |
| 16 | weed*[Title/Abstract] OR algae[Title/Abstract] OR fungi[Title/Abstract] OR fungus[Title/Abstract] |
| 17 | phytomedicine[Title/Abstract] |
| 18 | #8 OR #9 OR #10 OR #11 OR #12 OR #13 OR #14 OR #15 OR #16 OR #17 |
|  |  |
| **Embase** | |
| 1 | melanosis'/exp |
| 2 | (melanosis OR melasma OR chloasma OR freckle* OR melanoses OR melanism):ti,ab,kw |
| 3 | #1 OR #2 |
| 4 | 'medicinal plant'/exp |
| 5 | 'herbal medicine'/exp |
| 6 | 'plant medicinal product'/exp |
| 7 | 'phytotherapy'/exp |
| 8 | 'plant extract'/exp |
| 9 | (herb* OR plant OR plants OR botanical OR weed* OR algae OR fungi OR fungus OR phytomedicine):ti,ab,kw |
| 10 | #4 OR #5 OR #6 OR #7 OR #8 OR #9 |
| 11 | #3 AND #10 |
|  |  |
| **Web of Science** | |
| 1 | TI=(melanosis OR melasma OR chloasma OR freckle* OR melanoses OR melanism) |
| 2 | TI=(herb* OR plant OR plants OR botanical OR weed* OR algae OR fungi OR fungus OR phytomedicine OR phytotherapy) |
| 3 | #1 AND #2 |
|  |  |
| **The Cochrane Library** | |
| 1 | MeSH descriptor: [Melanosis] explode all trees |
| 2 | (melasma OR chloasma* OR freckle* OR Melanoses OR Melanism):ti,ab,kw |
| 3 | #1 or #2 |
| 4 | MeSH descriptor: [Plants, Medicinal] explode all trees |
| 5 | MeSH descriptor: [Herbal Medicine] explode all trees |
| 6 | MeSH descriptor: [Drugs, Chinese Herbal] explode all trees |
| 7 | MeSH descriptor: [Phytotherapy] explode all trees |
| 8 | MeSH descriptor: [Plant Extracts] explode all trees |
| 9 | (herb* OR plant OR plants OR botanical OR weed* OR algae OR fungi OR fungus OR phytomedicine):ti,ab,kw |
| 10 | #4 or #5 or #6 or #7 or #8 or #9 |
| 11 | #3 and #10 |


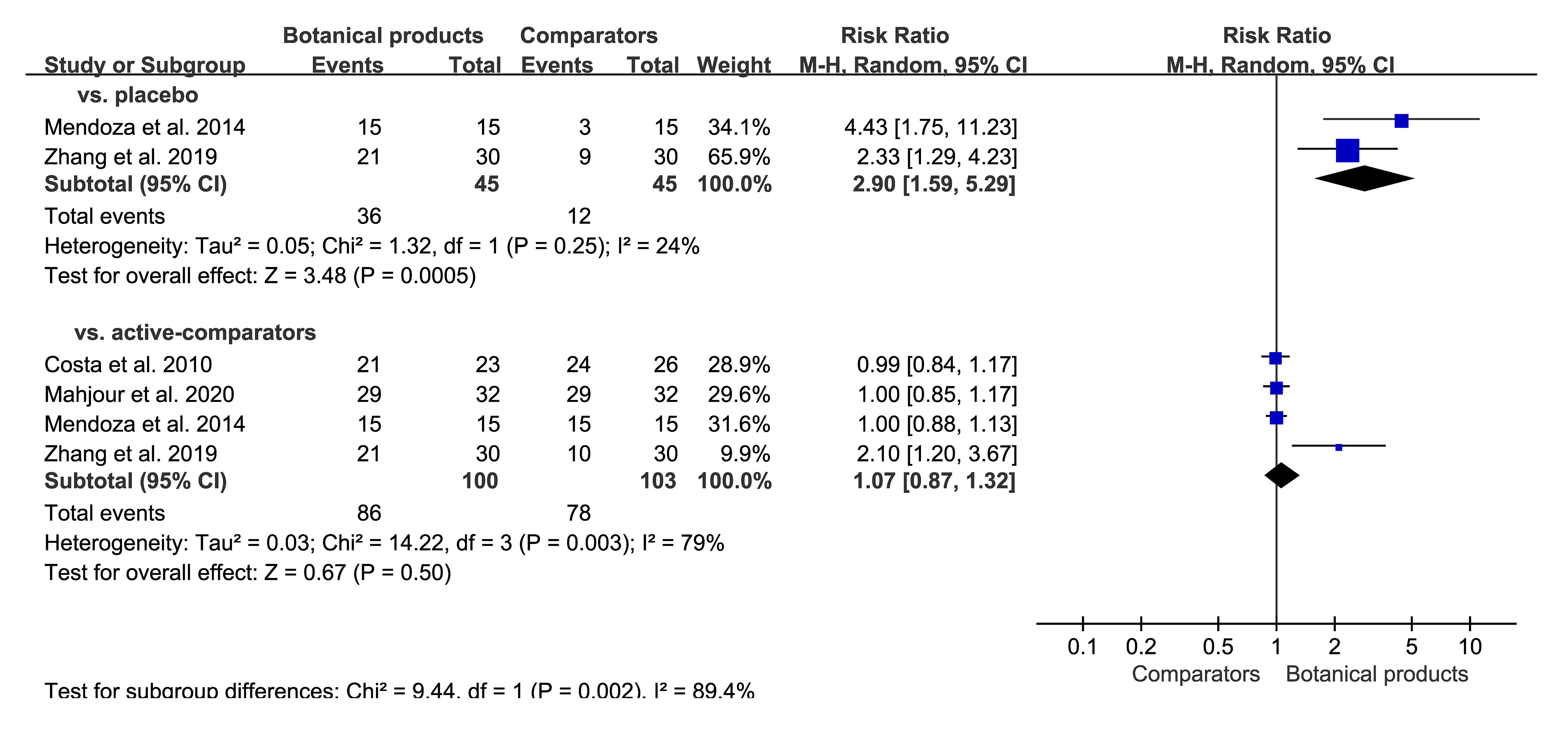


**Supplementary Figure 1** Forest plot depicting the risk ratio (RR) of outcome measure with self-evaluation in melasma patients receiving botanical products in randomised controlled trials (RCTs). Subgroup analysis was stratified according to comparators.


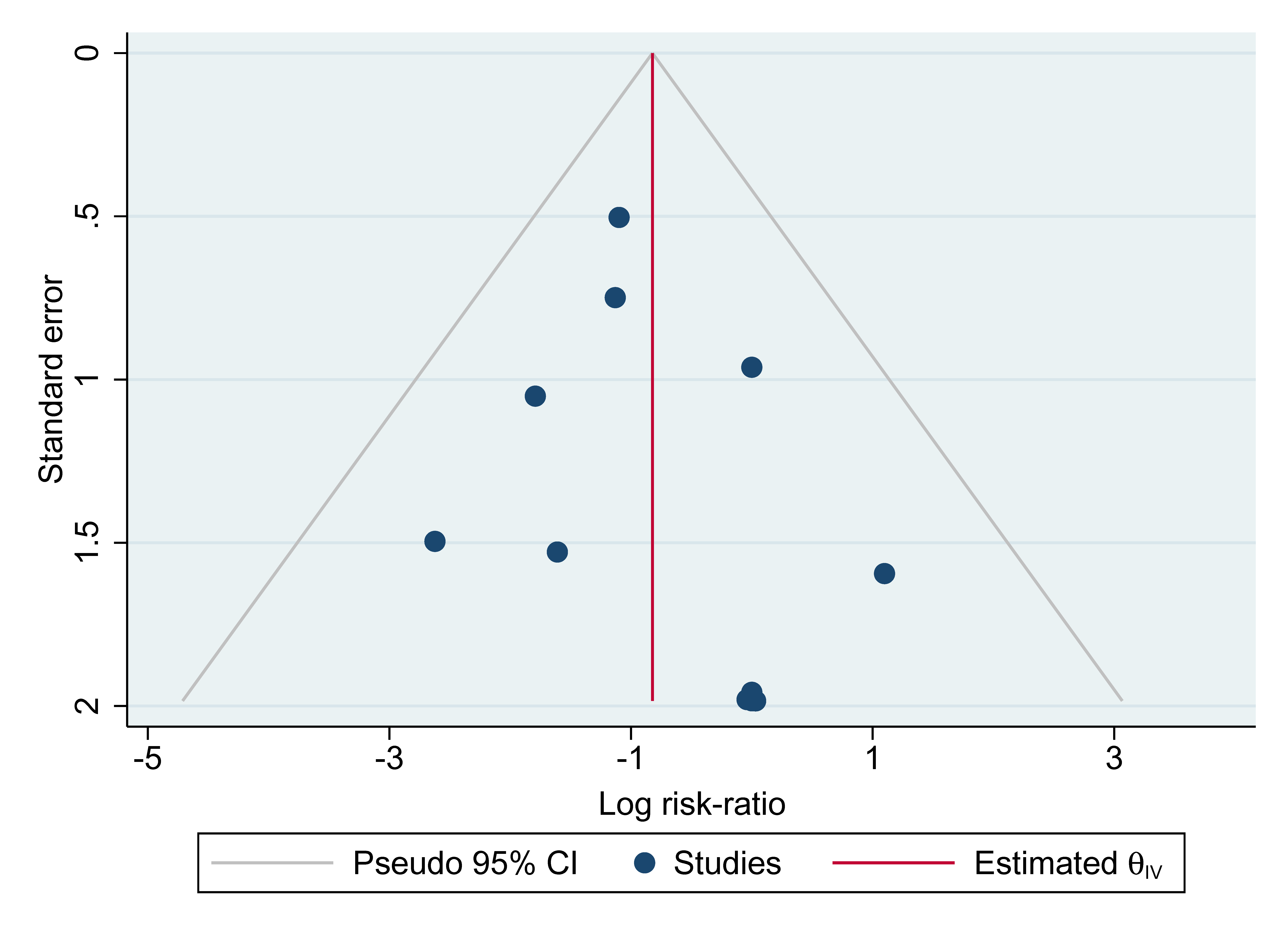


**Supplementary Figure 2** Funnel plot for randomised controlled trials (RCTs) reported safety outcome.
